# RSS-Based Localization and Mobility Evaluation Using a Single NB-IoT Cell†

**DOI:** 10.3390/s20216172

**Published:** 2020-10-29

**Authors:** Thomas Janssen, Rafael Berkvens, Maarten Weyn

**Affiliations:** IDLab-Faculty of Applied Engineering, University of Antwerp-imec, Sint-Pietersvliet 7, 2000 Antwerp, Belgium; thomas.janssen@uantwerpen.be (T.J.); rafael.berkvens@uantwerpen.be (R.B.)

**Keywords:** NB-IoT, LPWAN, localization, RSS, mobility

## Abstract

Low Power Wide Area Networks (LPWAN) have the ability to localize a mobile transmitter using signals of opportunity, as a low power and low cost alternative to satellite-based solutions. In this paper, we evaluate the accuracy of three localization approaches based on the Received Signal Strength (RSS). More specifically, the performance of a proximity, range-based and optimized fingerprint-based algorithm is evaluated in a large-scale urban environment using a public Narrowband Internet of Things (NB-IoT) network. The results show a mean location estimation error of 340, 320 and 204 m, respectively. During the measurement campaign, we discovered a mobility issue in NB-IoT. In contrast to other LPWAN and cellular technologies which use multiple gateways or cells to locate a device, only a single cell antenna can be used for RSS-based localization in NB-IoT. Therefore, we address this limitation in the current NB-IoT hardware and software by studying the mobility of the cellular-based 3GPP standard in a localization context. Experimental results show that the lack of handover support leads to increased cell reselection time and poor cell sector reliability, which in turn results in reduced localization performance.

## 1. Introduction

With the number of Internet of Things (IoT) devices that keeps growing rapidly, it has become indispensable to provide a location estimate of these devices in end user applications. For example, the location of mobile air quality sensors is essential when monitoring the air quality in a city. Oftentimes, the devices are battery-equipped and need to last for several years. Therefore, it is of utmost importance to localize them with as little energy as possible.

Traditional localization applications use a Global Navigation Satellite System (GNSS) in order to locate or track the position of a mobile device, with an accuracy of up to a few meters. However, satellite-based solutions have their limitations in the context of IoT. First, IoT devices often move between indoor and outdoor scenarios, e.g., an asset lying in a warehouse waiting to be transported. Since satellite signals do not penetrate well through walls, satellite-based solutions are not suitable in this situation. Second, a GNSS receiver consumes a significant amount of energy, reducing the expected battery lifetime from a few years to a few days or weeks, depending on the location update rate. Finally, a GNSS receiver is able to locate itself based on the Time of Flight (ToF) and constellation of available satellites. However, in many IoT applications, the position of the mobile device needs to be known at the network or application side, which consequently requires the device to send the estimated GNSS coordinate over a wireless network to the application, consuming even more energy.

In contrast to GNSS solutions, Low Power Wide Area Network (LPWANs) provide an energy-efficient solution to communicate short messages between a mobile device and an end user application. Moreover, the Base Station (BSs) of an LPWAN network can be used to estimate the location of a Mobile Terminal (MT). While it is unlikely that this approach will be as accurate as GNSS-based approaches, LPWAN localization is further encouraged in privacy sensitive applications. More specifically, sometimes it is not desired to know the exact location (i.e., within a few meters) of a person or a package. For a logistics company, for instance, it is usually sufficient to have a rough location estimate of an asset.

As LPWAN technologies often use star topologies, non-cooperative localization (NCL) schemes are mostly considered in LPWAN-based localization. In NCL schemes, end devices cannot directly communicate with other nearby end devices. In contrast, cooperative localization (CL) systems can lead to higher accuracies; however, they do not outweigh the increased device complexity and power consumption, which play a significant role in many LPWAN applications. For the same reason, it is often preferred to perform the localization in the cloud, instead of on the device itself.

LPWAN localization systems may be approached using various technologies such as Sigfox, LoRaWAN and Narrowband Internet of Things (Narrowband IoT (NB-IoT)). The focus in this research is on outdoor localization using a public NB-IoT network. An overview of the history of NB-IoT releases, as well as detailed specifications of both physical and data link layers of NB-IoT can be found in [1]. Released in 2016 by the Third Generation Partnership Project (3GPP), NB-IoT has several advantages over other technologies, making it one of the most popular LPWAN technologies. In contrast to Sigfox and LoRaWAN, the cellular-based NB-IoT technology operates in licensed frequency bands. Consequently, there are less duty cycle limitations and interference effects. Moreover, the use of existing Long Term Evolution (LTE) infrastructure enables providers to efficiently deploy a scalable NB-IoT network.

Release 14 of the 3GPP introduced Observed Time Difference of Arrival (OTDoA) in NB-IoT [2]. This downlink-based localization approach combines the Reference Signal Time Difference (RSTD) measurements between neighboring cells and a reference cell to estimate the location of a mobile User Equipment (UE). However, in order for this localization approach to work, all Evolved Node B (eNBs) need to be synchronized, which is a complex and expensive task for operators. Therefore, the OTDoA feature has not yet been implemented in many countries. On top of that, an OTDoA-capable UE requires the presence of highly accurate clocks, which is often not the case in low-cost IoT devices. Thus, there is a need for more energy-efficient, low-complexity and low-cost localization methods in NB-IoT.

In previous research, we performed the first real-world Received Signal Strength (RSS)-based outdoor localization experiments with NB-IoT [3]. It was found that only a single eNB antenna could be used to localize MT. This is in contrast to Sigfox and LoRaWAN, where multiple nearby gateways receive the message from the MT. In this paper, we extend our work with the following contributions. Firstly, we elaborate on the accuracy of RSS-based localization algorithms using a single NB-IoT cell. Secondly, we address the mobility issues of NB-IoT in a localization context. More specifically, we investigate the reason why only the serving cell can be used to locate a transmitter and we evaluate the cell sector reliability, as well as the cell reselection time.

The remainder of this paper is structured as follows. Section 2 provides an overview of recent outdoor localization studies using NB-IoT. In Section 3, the data sets used for both localization and cell reselection evaluation are described. Next, three optimized RSS-based localization algorithms are proposed in Section 4, namely proximity, range-based and fingerprint-based localization. Section 5 shows the setup to evaluate the cell reselection in NB-IoT. The results in terms of localization accuracy and mobility performance are shown in Section 6. Finally, the main observations regarding localization accuracy and mobility of NB-IoT are discussed in Section 7 and summarized in Section 8.

## 2. Related Work

Oftentimes, the preferred technology of a localization or tracking application highly depends on the requirements. In many IoT applications, energy efficiency, low cost and low complexity are given higher priorities than meter-level localization accuracy [4]. Therefore, LPWAN technologies such as Sigfox, LoRaWAN and NB-IoT are being used to estimate the location of a mobile device in a large-scale outdoor environment with minimal energy consumption [5,6].

Regardless of the technology, different localization approaches exist. In general, they can be classified into fingerprint-based, range-based, and Angle of Arrival (AoA)-based approaches. In fingerprint-based approaches, a new measurement is matched against earlier collected measurements. For example, Song et al. use Channel State Information (CSI) of NB-IoT for indoor pattern matching localization [7]. In ranging experiments, the signal strength or timing information from a MT to a BS is translated into an estimated distance using a path loss model [8]. In AoA-based approaches, one estimates the direction of the transceiver with respect to the gateway [9]. Recently, we proposed a multimodal localization framework, enabling to switch to an optimal localization method, as well as location-based seamless handover mechanisms [10].

While the worldwide deployment of public NB-IoT networks continues rapidly, little interest has been shown by operators to roll out the OTDoA feature in their networks. The core concepts of OTDoA, i.e., Narrowband Positioning Reference Signal (NPRS) and RSTD measurements, are very well explained in [11]. The implementation of this time-based localization feature requires highly accurate clocks in the UE and precise synchronization between the eNBs, leading to an increased complexity and cost of both UE and eNBs. For this reason, most performance evaluations of OTDoA-based positioning are carried out in the form of simulation studies [11,12,13,14]. Very recently, the first implementation of an OTDoA-capable NB-IoT chip was presented [15]. Measurements carried out in a laboratory setup result in RMS error of around 50 m. Important to note is the fact that due to the presence of a location server in an OTDoA-capable network, multiple eNBs can be used to estimate the location of a mobile UE. As an alternative to OTDoA, a 1-bit passive radar was used to reduce the complexity and energy consumption of the localization [16]. However, time-based localization methods are extensively studied in literature and are out of the scope of this paper.

Due to the high cost, high complexity and unavailability of OTDoA, RSS-based localization methods are being investigated. In order to improve the accuracy of outdoor RSS fingerprinting, the distance and RSS representation in ML algorithm should be optimized, as suggested in [17]. We carried out this optimization process for Sigfox in [18] and it was further analyzed by Anagnostopoulos et al. in [19]. Using an optimal parameter set in a *k* Nearest Neighbors (*k*NN) algorithm, a mean urban accuracy of 340 m is achieved. Although the *k*NN algorithm might seem very basic, it outperforms other Machine Learning (ML) algorithms such as Support Vector Machine (SVMs) in terms of localization accuracy and latency [20]. In [3], we conducted the first RSS-based experiments with NB-IoT. In this paper, we will further analyze the accuracy of a simple proximity algorithm, a range-based algorithm using several path loss models and an optimized fingerprint-based algorithm.

During the real-world localization experiments in [3], we experienced some mobility issues in the NB-IoT protocol. It was found that only the currently serving cell was reported to the UE, while neighboring cells in range were not reported. Obviously, this behavior is undesirable, since more participating eNBs result in higher localization accuracy [21]. According to Radnosrati et al., the number of reported cells is reduced to a minimum in order to minimize the signaling cost and support a large number of devices [22,23]. On the other hand, from 3GPP Release 16 onward, a UE may optionally report about strongest neighbors [1]. Many simulation studies use the LTE module in Network Simulator 3 (NS3) to simulate a real NB-IoT network [24]. However, by doing so, they do not face the aforementioned issue. Therefore, we chose to evaluate the performance of NB-IoT localization using real-world measurement data.

Another mobility issue in the NB-IoT protocol is the lack of handover support. Moon et al. explain the concepts of extended Discontinuous Reception (eDRX) and cell reselection of NB-IoT [25]. They acknowledge that cell reselection can only take place while in idle mode. Moreover, the cell selection process described in [23] suggests that only after failing to connect to the currently serving cell, the cell reselection process is initiated. Additionally, this issue introduces latency which can reach up to 10 s [26]. Furthermore, it is proven that when reselecting a cell more frequently, despite the increased energy consumption, the mobility performance increases significantly [25].

## 3. Outdoor NB-IoT Database

The database used in this research consists of two data sets. The first data set contains NB-IoT messages collected during an outdoor measurement campaign in the city of Antwerp, Belgium. This data set is used to evaluate the performance of RSS-based localization algorithms in an urban environment. The second data set is smaller and is used to evaluate the mobility of NB-IoT.

**Figure 1:**
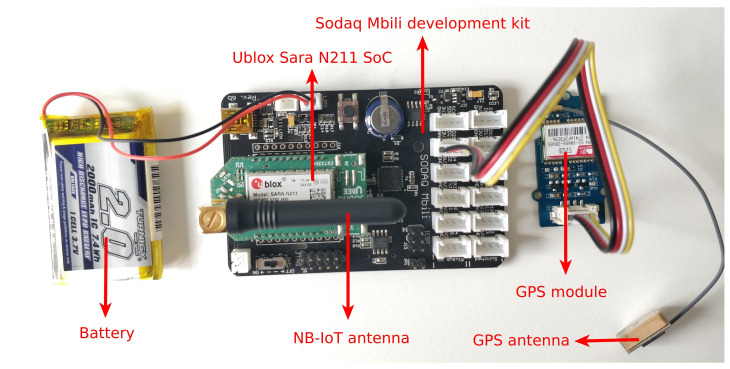
Composition of the User Equipment (UE) used to collect NB-IoT messages.

All NB-IoT messages are collected by sending uplink messages from a Ublox Sara N211 System on Chip (SoC) over a Release 13 NB-IoT network to a backend. Each message contains the Cell ID of and the RSS value to the responding eNB. By connecting a Global Positioning System (GPS) module to the UE, the current location (i.e., latitude, longitude, altitude) is added to the message. The GPS coordinate is used as a ground truth reference location. Therefore, in the following, we define the location estimation error as the geographical distance from the estimated location to the location provided by the GPS receiver. A picture of the battery-equipped UE with GPS is shown in Figure 1.

**Figure 2:**
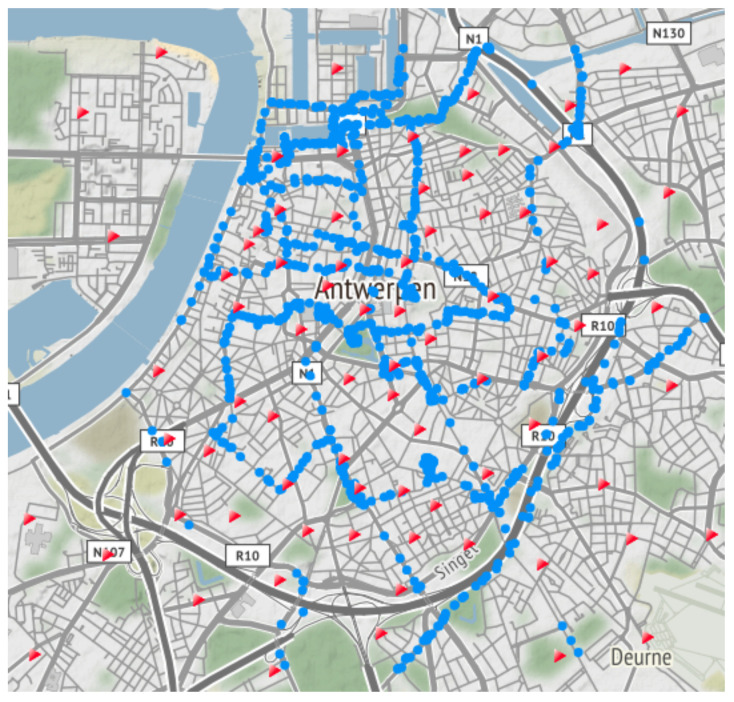
Map of the 53 ${\mathrm{km}}^{2}$ test environment in the city of Antwerp, Belgium, used to study the localization accuracy. The 1307 blue dots represent the GPS locations of the transmitter where a message was sent, while the 83 red triangles indicate the eNB locations.

Since we collected a large outdoor publicly available Sigfox and LoRaWAN data set in the city of Antwerp [27], we decided to perform the NB-IoT measurements in the same environment. In this way, we are able to compare the localization performance of the three most popular LPWAN technologies available on the market. The heterogeneous zone of interest covers an area of 53 ${\mathrm{km}}^{2}$ and is fully covered by a public NB-IoT Release 13 network, consisting of 83 eNBs. During the measurement campaign, we collected 1307 NB-IoT messages within the predefined zone, as shown in Figure 2.

**Figure 3:**
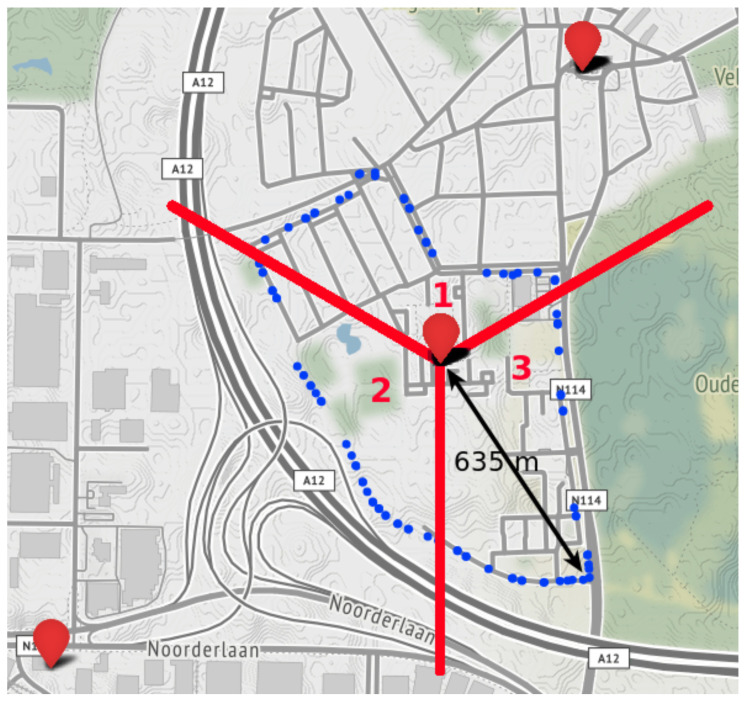
Map of the measurement campaign around a single eNB with three cell sectors, used to study the mobility. The 95 blue circles represent the GPS locations of each uplink transmission. The sector boundaries of each cell of the eNB are shown in red.

A smaller data set is collected in northern Antwerp. In order to evaluate the mobility issues addressed in Section 1, we want to analyze the performance of cell selection and reselection in NB-IoT. The eNB centered around the track of our measurement campaign consists of three directional antennas, with each cell covering a sector of 120${}^{{}^{\circ}}$. Note that other configurations such as four antennas separated 90${}^{{}^{\circ}}$ exist. To evaluate the handover between two antennas or cells, we collected 95 messages by moving counterclockwise around the eNB at a constant speed of per-mode=symbol 7 km/h and an update rate of 5 s. Figure 3 depicts a map of the uplink messages, together with the cell sector boundaries.

## 4. RSS-Based Localization Algorithms

In this section, three RSS-based methodologies to locate a mobile UE are presented. First, a simple proximity algorithm is presented. Second, we modify a range-based algorithm, which uses a path loss model in combination with the sector information of the received eNB. Finally, we create a fingerprinting database and optimize the hyperparameters of a *k*NN algorithm.

### 4.1. Proximity Localization

In a proximity-based localization algorithm, the location of the eNB with the strongest link to a mobile UE is used as the estimated location of that UE. This basic yet efficient RSS-based algorithm can already satisfy the requirements of several localization or tracking applications, such as logistics use cases and asset tracking. However, the accuracy highly depends on the base station density and type of environment.

### 4.2. Range-Based Localization

In RSS-based ranging approaches, the RSS from a UE to a specific eNB is translated into a distance by means of a propagation channel model or path loss model. Most path loss models can be customized by setting parameters, depending on the environment. Given the urban nature of our large-scale environment, we set the height of the mobile station $h_{MS}$ to 2 m and the height of the base station $h_{BS}$ to 27 m. The carrier frequency *f* varies between 800 and 900 MHz and the transmission power is set to 14 dBm.

In this paper, we evaluate the performance of four urban path loss models. The Hata model is an empirical path loss model based on the widely used model of Okumura [28], and the path loss $L_{P}$ of the urban variant can be characterized as follows:

(1) $$L_{P,Hata}=A+B\log_{10}\left(d\right),$$

with

(2) $$\begin{array}{cc} A & =69.55+26.16\log_{10}\left(f\right)-13.82\log_{10}\left(h_{BS}\right)-a\left(h_{MS}\right), \end{array}$$

(3) $$\begin{array}{cc} B & =44.9-6.55\log_{10}\left(h_{BS}\right), \end{array}$$

where

(4) $$a\left(h_{MS}\right)=3.2*{\left[\log_{10}\left(11.75*h_{MS}\right)\right]}^{2}-4.97.$$

The European Cooperation in Science and Technology (COST) received funding to extend the Hata model, resulting in the COST-231 model [28]:

(5) $$L_{P,COST-231}=A+B\log_{10}\left(d\right)+C,$$

with

(6) $$\begin{array}{cc} A & =46.3+33.9\log_{10}\left(f\right)-13.28\log_{10}\left(h_{BS}\right)-a\left(h_{MS}\right), \end{array}$$

(7) $$\begin{array}{cc} B & =44.9-6.55\log_{10}\left(h_{BS}\right),\;\mathrm{and} \end{array}$$

(8) $$\begin{array}{cc} C & =3. \end{array}$$

Next, two empirical outdoor path loss models based on the 3GPP Spatial Channel Model (SCM) were proposed by the IEEE TGah group, which standardizes IEEE 802.11ah. Although the original models were devised for LTE, they have been transformed for use in sub-gigahertz (sub-GHz) frequency bands [29]. The first model targets a macro Line-of-Sight (LoS) deployment scenario (further referred to as *AH-macro*) and can be expressed as in the following equation:

(9) $$L_{P,AH-macro}=8+36.7\log_{10}\left(d\right).$$

A second 3GPP model targets a pico deployment scenario (further referred to as *AH-pico*) and can be characterized using the formula:

(10) $$L_{P,AH-pico}=23.3+36.7\log_{10}\left(d\right)+c_{pico},$$

with

(11) $$c_{pico}=21\log_{10}\left(f/900\right).$$

**Figure 4:**
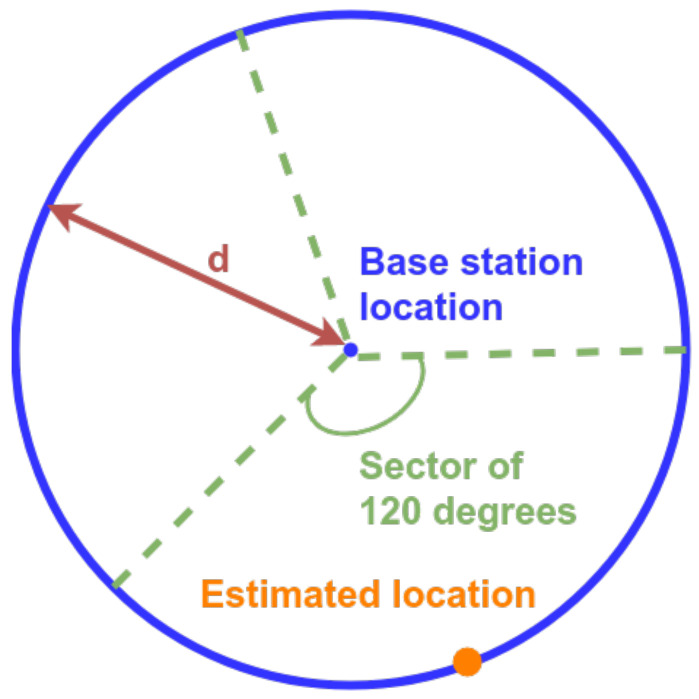
Visualization of the RSS-based ranging algorithm with sector information. A single receiving NB-IoT base station antenna covers a sector of 120 degrees. The RSS from that antenna is translated into a distance *d*. The location of the UE is estimated to be at the center of the resulting arc.

Given the mobility issues addressed in Section 1, it is not possible to retrieve information about multiple neighboring cells in NB-IoT. In other words, only the antenna of the serving eNB can be used to estimate the location of a UE. As a consequence, well known ranging localization approaches such as triangulation or multilateration cannot be applied. Therefore, we introduce a novel algorithm that estimates the position of a UE based on the combination of the serving cell and sector information. First, we estimate the distance *d* between the UE and eNB using one of the four previously presented path loss models. This results in a circle around the eNB with a radius equal to *d*, representing all possible UE locations. With the aim to further improve the localization accuracy, we take into account the azimuth of the directional antenna of the serving cell, which covers a sector of 120 ${}^{{}^{\circ}}$. Thus, the antennas provide a spatial filter, eliminating location estimates located outside a given sector. As we assume the location of the UE on the resulting arc to be Gaussian distributed, the final location estimate is set to the center of the arc, as illustrated in Figure 4.

### 4.3. Fingerprint-Based Localization

Fingerprinting is a localization technique that is well established in indoor localization applications. The reason for this is that the first phase, i.e., the building of a training database, requires a significant amount of time and effort. However, recent crowd-sourced database initiatives can tackle this problem. Therefore, in this paper, we optimize ML-based fingerprinting algorithm for localization in an outdoor environment.

**Figure 5:**
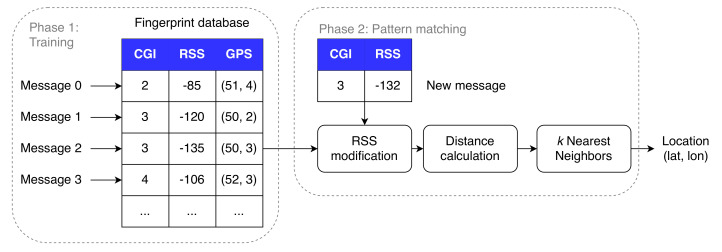
Procedure of the fingerprint-based *k*NN algorithm.

As in many ML-based approaches, the localization data set described in Section 3 is split into 70% training messages, 15% test messages and 15% validation messages. The actual fingerprinting technique is split into two phases, as visualized in Figure 5. The first and offline phase consists of collecting training messages into a fingerprint database. Each entry of the fingerprint database consists of the RSS value to the serving eNB, together with the Cell Global Identity (CGI) and GPS coordinate of the ground-truth location. Consequently, in the second and online phase, a test fingerprint is being matched to the earlier collected fingerprints, stored in the training database. Using the *k*NN algorithm, we calculate the distance between the test sample and each training sample in signal space. Afterwards, the location of the UE is estimated by computing the centroid of the ground truth locations of the *k* nearest neighbors. Additionally, we optimize several *k*NN-based algorithms in this work. In brief, we iterate over four different RSS representations, 31 distance metrics and several values of *k*, similar to what we did in [18]. After the most optimal parameter configuration is found, we validate our results using the messages in the validation data set.

## 5. NB-IoT Mobility Evaluation Setup

In traditional RSS-based localization algorithms, the position of MT is determined by combining the estimated distances to multiple base stations in a multilateration algorithm. For example in LTE, the location of a UE is estimated based on timing information to multiple eNBs, leading to an increased localization accuracy. Similarly, an OTDoA-capable UE is able to request information from neighboring cells, due to the presence of a location server in the OTDoA-capable NB-IoT network. However, only a single (serving) cell of an eNB can be used for RSS-based localization in NB-IoT [3]. Therefore, in this paper, we study the mobility of NB-IoT in a localization context. In order to assess the performance of the cell reselection process, we collected messages at a constant velocity and update rate around a single eNB with three cells, as discussed in Section 3 and shown in Figure 3. The azimuths of the three directional antennas are separated 120 degrees, pointing to 0${}^{{}^{\circ}}$, 120${}^{{}^{\circ}}$ and 240${}^{{}^{\circ}}$. The trajectory around the eNB was chosen with the objective to force a handover between each cell of the eNB. Consequently, we are able to evaluate two mobility parameters:

- Sector reliability, i.e., whether the UE is located inside the correct sector of the serving cell.
- Cell reselection time, i.e., how long it takes to switch to another antenna.

## 6. Results

During the large-scale RSS-based localization experiments, we discovered a mobility issue in the NB-IoT protocol. In this section, we first show the results of the localization algorithms in terms of location estimation error. Consequently, the mobility of NB-IoT is evaluated in terms of sector reliability and cell reselection time.

### 6.1. RSS-Based Localization Accuracy

The proximity algorithm results in a mean location estimation error of 340 m and a median of 294 m. In many applications, these rough location estimates can be sufficient, especially when the cost and complexity of the localization needs to be reduced to a minimum.

To optimize the ranging algorithm for our test environment, several urban path loss models have been evaluated. As shown in Table 1, all path loss models yield similar location estimation errors. The 3GPP AH-macro model yields the smallest mean and median localization error of 320 m and 259 m, respectively. Although the ranging algorithms perform only slightly better than the proximity algorithm, one needs to keep in mind that only a single eNB antenna sector is used to estimate the position of the mobile node.

Finally, a fingerprint-based *k*NN algorithm is evaluated and optimized. As suggested in [17], different RSS representations and distance metrics are evaluated, along with a parameter sweep of *k*, which is the amount of neighbors considered during the matching phase. In general, the mean estimation errors vary from 184 m to 207 m. In contrast to previous research, the lineal (i.e., positive and normalized) RSS representation yields the smallest location estimation error. In combination with the Pearson $\chi^{2}$ distance metric, the smallest location estimation error of 184 m is achieved for the test set. After iterating over different values of *k*, it appears that using the ground truth reference locations of the k=2 nearest neighbors results in the highest localization accuracy.

**Figure 6:**
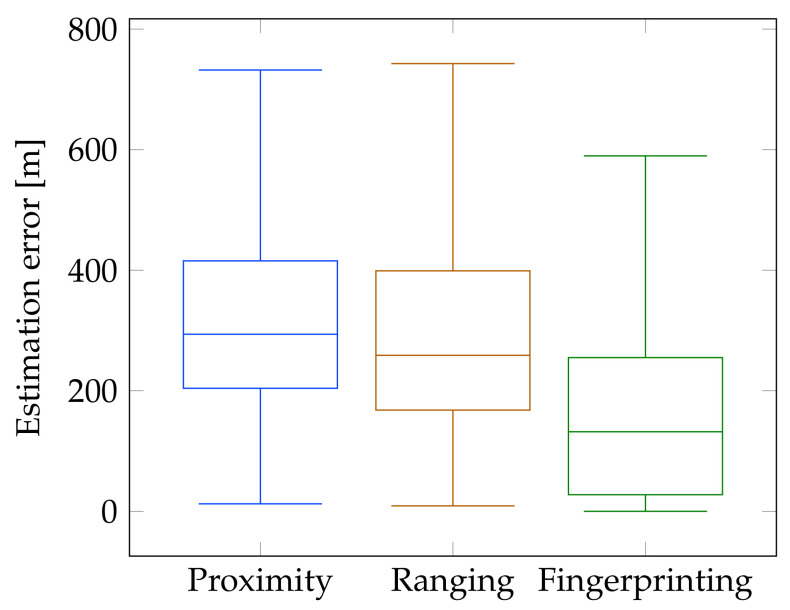
Location estimation errors for every outdoor RSS-based localization algorithm when using a single NB-IoT base station antenna. The left box plot shows the proximity algorithm results. The box plot in the middle shows the errors of the ranging algorithm with the 3GPP AH-maco path loss model. The right box plot shows the estimation errors of the validation fingerprinting data set using the optimal parameter configuration.

The location estimation errors from the test data set are validated by running the algorithm with the optimal parameter set again with unseen data from the validation data set. This yields a mean and median location estimation error of 204 m and 132 m, respectively. Moreover, 95% of all measurements result in a localization error under 679 m. The location estimation errors for the outdoor proximity, ranging and fingerprinting algorithms are summarized in Figure 6.

### 6.2. Mobility Evaluation

**Figure 7:**
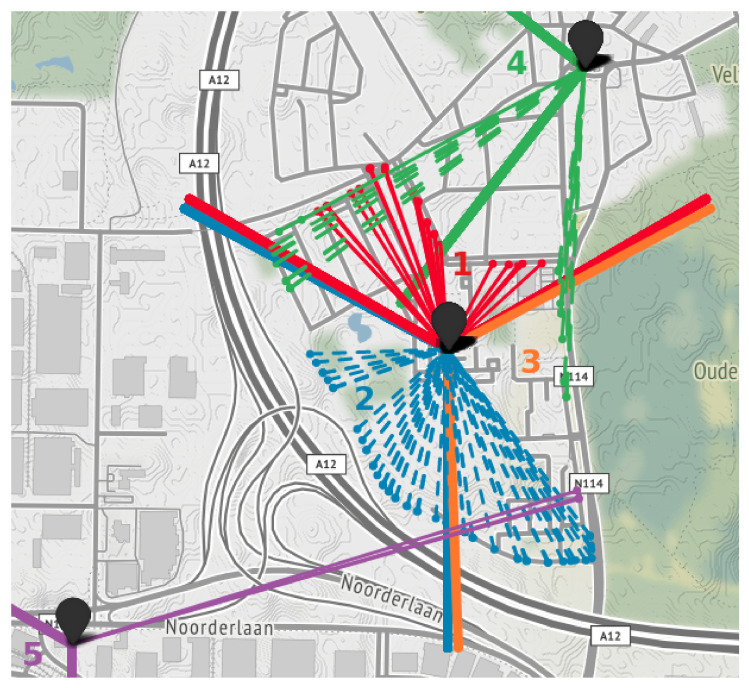
Visualization of NB-IoT cell reselection when moving at a constant speed around eNB with three cell sectors. The color and pattern of the line connecting the GPS location of the UE to the eNB indicates the currently serving cell. The cell sector boundaries are indicated with thicker lines.

Figure 7 visualizes the connections between every transmission location and the currently serving cell, as well as the respective cell sector boundaries. Starting at the north side and moving counterclockwise, the first UE positions (i.e., red dots) are located within sector 1, which is the sector of the serving cell. When the UE moves outside sector 1, the cell reselection takes place and in most cases the adjacent cell corresponding to sector 2 becomes the serving cell. After leaving sector 2, the UE remains connected to the cell related to sector 2 and never connects to the cell covering the area of sector 3. Instead, we observe serving cells from other eNBs for short amounts of time (i.e., cell sectors 4 and 5). Thus, only two out of three cells of the center eNB are used.

The time to select another serving cell varies from 51 s to 104 s, with a mean cell reselection time of 73 s. Within these periods, it is not possible to send NB-IoT messages. Furthermore, the UE remains connected to the cell covering sector 2 for more than four minutes after leaving that sector, without ever connecting to the cell corresponding to sector 3.

## 7. Discussion

Large-scale outdoor localization applications have been relying on GNSS-based solutions for years. However, with the emergence of IoT devices, we observe a growing need for alternative localization solutions that fulfill the requirements of long range and low power consumption. LPWANs provide a modern solution for both communication and localization of IoT devices. It is important not to look at GNSS-based and LPWAN-based solutions as competitors. In contrast, they can be complementary to each other, leading to combined positioning solutions such as Ubiscale (https://ubiscale.com/geo-iot/).

While OTDoA in NB-IoT networks promises high-level accuracy, the technology is still in its infancy. As synchronizing eNBs in an NB-IoT network is a challenging and costly task, few operators already deployed this feature in their networks. Moreover, a location server is required in an OTDoA-capable network, which reports on neighboring cells. Furthermore, a highly accurate clock required in the UE leads to additional device complexity, which is often not desired.

In this work, RSS-based localization has been studied using real-world NB-IoT measurements in a city-scale environment. Three RSS-based GPS-less algorithms are evaluated in terms of localization accuracy. The mean location estimation error varies from 340 m with a basic proximity algorithm to 204 m with an optimized fingerprinting approach. Set side by side, our previous research using multiple Sigfox base stations in the same environment resulted in a mean location estimation error of 340 m in the most optimal fingerprinting approach [18]. Moreover, we studied RSS-based localization with LoRaWAN, which coincidentally lead to an accuracy of 340 m as well, using another fingerprinting approach and multiple base stations [20]. Even though there are more NB-IoT base stations than Sigfox and LoRaWAN base stations located in the test environment, only a single base station antenna was used per measurement in the NB-IoT experiments. Contrarily, in the LoRaWAN experiments, up to 10 gateways were taken into account to estimate the location of the transmitting device. In the Sigfox experiments, the maximum number of receiving gateways increases to 48. From this point of view, the ability to reach multiple NB-IoT base stations promises to increase the localization accuracy significantly, outperforming other LPWAN technologies.

While the proximity algorithm results in a mean accuracy of 340 m, the localization accuracy of the RSS-based ranging algorithm increases only slightly to 320 m. This result can be explained by discussing three limitations of the proposed ranging algorithm. First, estimating the path loss in an urban but heterogeneous environment is not straightforward. Therefore, finding an optimal path loss model is a challenging task. Even if the path loss model estimates all distances very well, the average localization error highly depends on the sector size. This is due to the fact that all locations of UEs sending from within a certain cell sector and distance *d* to the eNB are estimated at the same centered location of that sector. A second limitation in the ranging algorithm is the fact that only a single directional cell antenna can be used to estimate the position of a UE. This is in contrast to Sigfox and LoRaWAN, where multiple receiving gateways yield more accurate results by performing multilateration. This argument indicates the growing need for multiple receiving cells in NB-IoT. Finally, the last limitation involves the issue of cell sector reliability, which is why we carried out a separate analysis of the mobility issues in an NB-IoT localization context.

Fingerprint-based localization approaches all share one major disadvantage: creating and maintaining a large-scale outdoor fingerprint database requires a significant amount of time and effort. For this reason, outdoor fingerprinting is not that popular as when compared to outdoor ranging or indoor fingerprinting. However, this issue is being tackled by crowd-sourcing initiatives such as *TheThingsNetwork* (https://www.thethingsnetwork.org/), which enable efficient worldwide collection of training data through sensors, chip sets and even smartphones. On the other hand, fingerprint-based approaches have several benefits over others. In contrast to ranging, the locations of the base stations do not have to be known. Furthermore, when collecting training data, the particularities of the signal strength are stored in the fingerprint database. For this reason, the proposed fingerprinting algorithm consistently yields the highest localization accuracy.

In order to evaluate the localization accuracy of an RSS-based fingerprinting approach, the parameters of a *k*NN algorithm are optimized. While in a similar study, the exponential RSS representation led to the highest localization accuracy [18], the best results in this study were obtained using the lineal (i.e., positive and normalized) RSS representation. Furthermore, the optimal parameter set can be expressed by the Pearson $\chi^{2}$ distance and k=2. Since the 1307 measurements in the fingerprinting data set are spread over an area of 53 ${\mathrm{km}}^{2}$, the distance between each measurement in the training set is quite large, which in turn leads to a smaller value of nearest neighbors *k*. Finally, the optimal parameter configuration is validated by running the *k*NN algorithm with unseen data, leading to a mean and median location estimation error of 204 m and 132 m, respectively.

During the collection of real-world measurement data for the localization study, we encountered some mobility issues in the NB-IoT protocol. Therefore, we highlight these issues in this work and analyze the impact on the localization performance. One major problem we faced was the inability to use information of neighboring cells in our localization algorithms. While in LTE, this feature has been implemented for years, only the serving cell can be used for RSS-based localization in NB-IoT. On the one hand, recent studies speculate that this limitation might be on purpose, in order to increase scalability and decrease signaling overhead and power consumption [22,23]. On the other hand, some speculate the feature will be included in a future release of NB-IoT [1]. Either way, multiple neighboring cell reports can definitely increase the accuracy of RSS-based localization. Therefore, the reception of multiple cells will be simulated in future work.

The results of the mobility analysis in Figure 7 demonstrate the cell reselection process of NB-IoT. When moving at a constant speed of per-mode=symbol 7 km/h, the cell reselection process took 73 s on average. Important to note is that this cell reselection time depends on both the velocity of the UE and cell reselection parameters configured by the operator. Decreasing the update rate of the cell reselection increases battery lifetime but is unfavorable from a localization perspective, as this introduces latency and reduced location update rates. Additionally, we investigated the cell sector reliability. After each cell reselection process, most of the messages are located within the sector of the new serving cell. However, it is worth discussing two peculiarities that can be observed from Figure 7. First, the UE remains connected to the same serving cell after moving outside the serving cell sector. Second, serving cells from eNBs located further away are reported, especially after losing the connection to the currently serving cell. The first issue might arise when the connection to the currently serving cell remains stronger than the connection to an adjacent cell, e.g., due to multipath or interference effects. The second issue can be caused by side and back lobes of directional antennas, which seem to establish a better connection for a short amount of time. Furthermore, despite being located in the serving cell sector, the UE might select a stronger cell from a nearby eNB if there is no more LoS to the current eNB. Thus, in line with the ideas of Moon et al. [25], it can be concluded that the performance of the cell reselection process in the NB-IoT technology should be improved.

## 8. Conclusions

The cellular-based NB-IoT technology connects a plethora of devices to the IoT. Consequently, there is an urgent need to localize such devices in an energy-efficient way. While OTDoA is introduced in 3GPP Release 14, few operators deployed this localization feature in their networks, given the cost and complexity to synchronize eNBs. Therefore, we investigated three RSS-based localization approaches, providing an energy-efficient solution at low cost and low complexity. Experiments carried out in a large-scale urban environment led to mean location estimation errors ranging from 340 m in a basic proximity algorithm to 204 m in an optimized fingerprinting algorithm. Traditional multilateration approaches could not be applied, since with current hardware and software only the serving cell antenna can be used to locate a mobile UE. In order to address this issue, we studied the mobility of NB-IoT in a localization context. Due to unpredictable antenna patterns and environmental influences, a UE might connect to a cell while not located inside the theoretical sector of that cell. Finally, this poor cell sector reliability and the long cell reselection times indicate the need for proper handover support and observation of multiple cells in NB-IoT networks.

## Figures and Tables

**Table 1:** Location estimation errors for different urban path loss models of an RSS-based ranging algorithm using a single NB-IoT antenna and cell sector.

Path Loss Model	Mean Error [m]	Median Error [m]	95th Percentile [m]
Hata	325	272	783
COST-231	327	276	780
**3GPP AH-macro**	**320**	**259**	**790**
3GPP AH-pico	330	279	776
